# Synchronous bilateral phyllodes tumors with discordant grades and false-positive axillary ^18^F-FDG PET-CT uptake: a case report and literature review

**DOI:** 10.3389/fonc.2026.1825190

**Published:** 2026-04-20

**Authors:** Tianle Yu, Ruiyong Li, Chunyu Cai

**Affiliations:** The Affiliated Hospital of Yanbian University (Yanbian Hospital), Yanji, China

**Keywords:** 18F-FDG PET-CT, axillary lymph nodes, breast, case report, Phyllodes tumor, synchronous bilateral

## Abstract

**Rationale:**

Phyllodes tumors are uncommon fibroepithelial breast neoplasms with variable biologic behavior. Synchronous bilateral phyllodes tumors are exceptionally rare, and the interpretation of ^18^F-fluorodeoxyglucose positron emission tomography/computed tomography (^18^F-FDG PET-CT) may be confounded by tumor necrosis and inflammation, leading to false-positive nodal staging.

**Patient concerns:**

A 44-year-old Korean woman presented with a rapidly enlarging, painful, ulcerated left breast mass for 3 months. She had no nipple discharge, fever, or weight loss. A right breast lesion was clinically occult.

**Diagnoses:**

Ultrasound showed a right breast hypoechoic nodule (BI-RADS 4A) with scant vascularity and an elastography score of 2, and a giant heterogeneous hypervascular left breast mass with enlarged axillary lymph nodes and an elastography score of 4. ^18^F-FDG PET-CT revealed intense uptake in the left breast mass (SUV_max_ 11.5) and markedly FDG-avid ipsilateral axillary nodes (SUV_max_ 9.0), suspicious for metastasis; mild uptake was seen in the right breast nodule (SUV_max_ 1.7) without definite distant metastasis. Core needle biopsy supported a fibroepithelial neoplasm favoring phyllodes tumor. Final pathology confirmed a left borderline phyllodes tumor and a right benign phyllodes tumor; all 21 axillary lymph nodes showed reactive hyperplasia with sinus histiocytosis and no metastasis.

**Interventions:**

After multidisciplinary review, the patient underwent excision of the right breast mass with frozen-section margin assessment, left total mastectomy including the nipple-areola complex and ulcerated skin to achieve negative margins, and level I-II axillary lymph node dissection because sentinel lymph node biopsy was considered unreliable in the setting of massive ulceration and severe axillary edema.

**Outcomes:**

Recovery was uneventful. Ceftriaxone sodium 2.0 g was given intravenously once daily from 1 day before surgery through postoperative day 5. No wound infection, seroma, fever, or unexpected adverse events occurred. At 1 month, CA125, CA15-3, and CA19–9 had normalized.

**Lessons:**

In phyllodes tumors, marked axillary FDG avidity may reflect reactive inflammation rather than true nodal metastasis, particularly in giant ulcerated tumors with necrosis. Management should emphasize complete excision with negative margins and cautious axillary surgery guided by clinicopathologic correlation.

## Introduction

Phyllodes tumors (PTs) are rare fibroepithelial breast neoplasms, accounting for approximately 0.3%–1% of all primary breast tumors and 2%–3% of fibroepithelial breast lesions (1). According to the 2019 World Health Organization (WHO) framework, PTs are classified as benign, borderline, or malignant based on stromal cellularity and atypia, mitotic activity, stromal overgrowth, and the character of the tumor border (2). While most PTs follow a relatively indolent course, borderline and malignant tumors are associated with higher local recurrence risk and, particularly for malignant PT, potential for distant metastasis (1, 3). Clinically, PTs typically present as a unilateral, painless, firm breast mass that may demonstrate rapid interval growth (1). Synchronous bilateral PTs are exceptionally uncommon, and published experience remains limited largely to case reports (4). Preoperative discrimination between fibroadenoma and benign PT is often challenging because of overlapping imaging and core-biopsy features (1, 6). Although ^18^F-FDG PET-CT is used in oncologic staging, its role in PTs is not well standardized; moreover, FDG avidity may be confounded by inflammation and necrosis, which can contribute to false-positive interpretations in ulcerated or infected giant tumors (1, 7). The cornerstone of treatment is complete surgical excision with negative margins to reduce recurrence; however, recurrence risk varies by histologic grade, margin status, and surgical approach (3, 5). Axillary management should be individualized because nodal metastasis is rare, whereas reactive lymphadenopathy may complicate operative decision-making (1, 16). Herein, we report synchronous bilateral PTs in a 44-year-old woman, with a giant ulcerated borderline tumor in the left breast and a benign tumor in the right breast. This case highlights the diagnostic pitfall of marked axillary ^18^F-FDG uptake mimicking metastasis and underscores the importance of clinicopathologic correlation in distinguishing reactive lymphadenopathy from malignant spread.

## Case presentation

### Clinical presentation

A 44-year-old Korean woman presented to the Department of Breast Surgery at Yanbian University Hospital on May 18, 2025, with a palpable mass in the left breast that had been present for 3 months. The mass was initially painless and stable in size but enlarged rapidly over the preceding month, accompanied by localized pain and spontaneous skin ulceration. She denied nipple discharge, fever, or unintentional weight loss. She was a homemaker and reported no tobacco or alcohol use. Her medical history was notable only for wrist surgery following trauma 20 years earlier. She reported no family history of breast or ovarian cancer and had no chronic comorbidities, including hypertension, diabetes mellitus, or cardiovascular disease.

The key diagnostic and therapeutic events are summarized in the following timeline.

**Figure 1:**
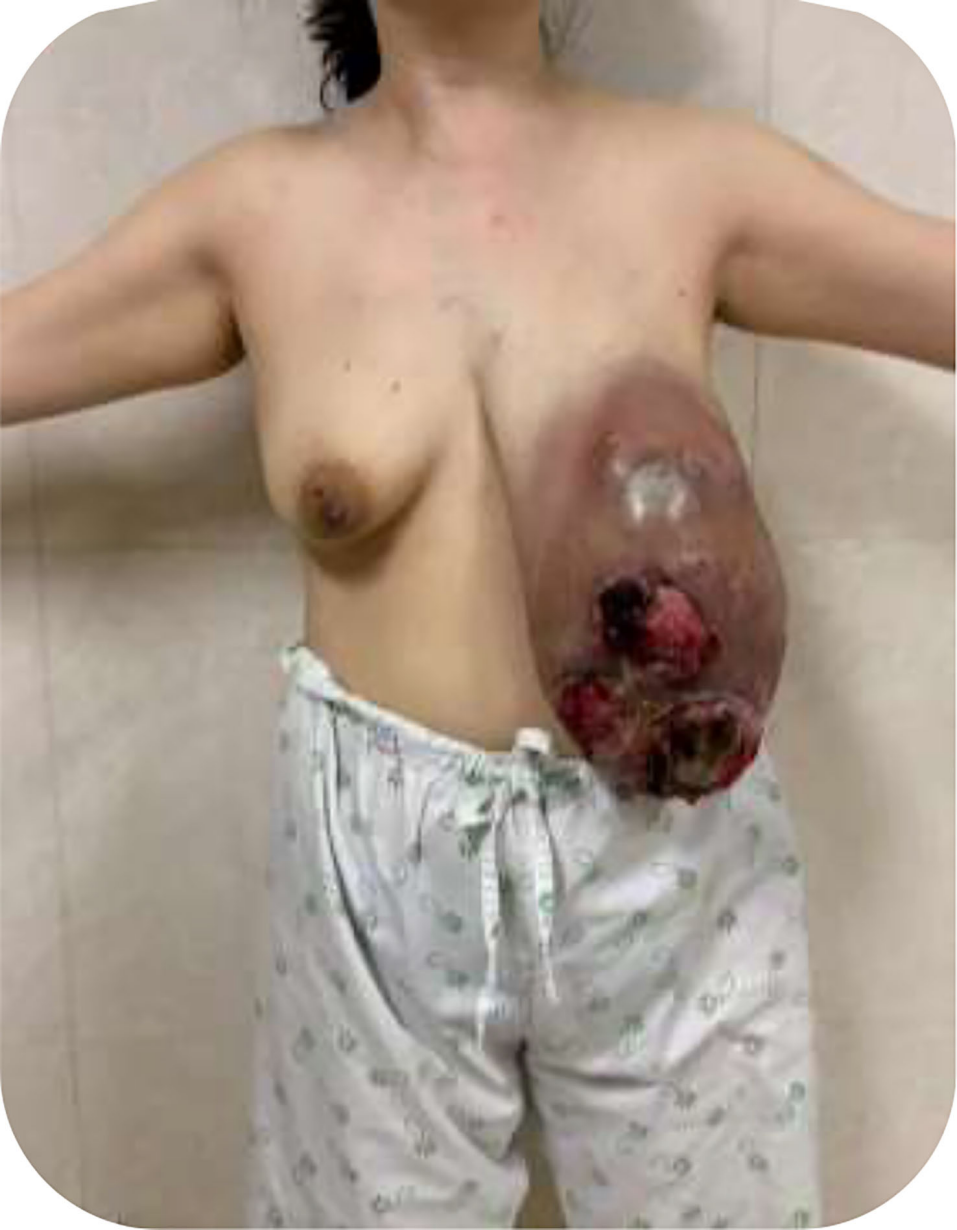
Preoperative clinical photograph of the left breast showing a giant, tense mass with multiple necrotic ulcerations.

On admission, vital signs were within normal limits. Physical examination revealed marked breast asymmetry. In the left breast, a giant, firm mass occupied nearly the entire breast, measuring approximately 24 × 17 × 15 cm. The overlying skin was erythematous and edematous with a peau d’orange appearance. Three discrete ulcerations with necrotic bases were present, measuring approximately 10 × 7 cm, 10 × 7 cm, and 5 × 5 cm. Tumor mobility relative to the chest wall was markedly limited, largely attributable to tumor bulk. The right breast showed no skin changes or nipple retraction. Palpation was initially noncontributory because of the dominant contralateral lesion; however, a discrete nodule was subsequently identified on imaging. Palpation of the axillary and supraclavicular basins was limited by local edema and body habitus, precluding reliable clinical assessment for lymphadenopathy (**Figure 1**).

### Diagnostic workup

Preoperative laboratory testing on May 19, 2025, demonstrated a pronounced systemic inflammatory response in the setting of extensive tumor necrosis and ulceration. C-reactive protein was 165.7 mg/L (reference <10 mg/L), serum amyloid A was 368.7 mg/L, and interleukin-6 was 61.9 pg/mL. Serum tumor markers were elevated, including CA-125 155.0 U/mL, CA15-3 77.2 U/mL, and CA19-9 52.8 U/mL. Because the initial differential diagnosis included locally advanced breast carcinoma, primary breast sarcoma, and phyllodes tumor, these tumor markers were obtained as part of the preoperative diagnostic evaluation rather than as PT-specific biomarkers. In this clinical context, their elevation was interpreted as most likely secondary to extensive tissue necrosis and the associated systemic inflammatory response, rather than reflecting PT-specific biologic activity. Perioperative infection-related and hematologic indices are summarized in **Table 1**: on admission, the white blood cell count was 14 × 10^9/L, neutrophils were 10 × 10^9/L, procalcitonin was <0.1 ng/mL, and hemoglobin was 98 g/L; immediately preoperatively, the corresponding values were 12 × 10^9/L, 8.5 × 10^9/L, <0.1 ng/mL, and 95 g/L. Given the ulcerated necrotic surface and the risk of secondary infection, ceftriaxone sodium 2.0 g by intravenous drip once daily was administered from 1 day before surgery through postoperative day 5. Mild postoperative anemia was documented on postoperative day 1 (hemoglobin 93 g/L), attributed to perioperative blood loss and inflammation, and improved to 109 g/L by discharge.

**Table 1:** Perioperative infection-related and hematologic indices.

Time point	WBC (×10^9/L)	NEU (×10^9/L)	PCT (ng/mL)	Hb (g/L)
Admission (May 18, 2025)	14	10	<0.1	98
Pre-op (May 21, 2025)	12	8.5	<0.1	95
Post-op day 1 (May 23, 2025)	11	8.7	<0.1	93
Discharge (June 3, 2025)	8	8	<0.1	109

**Figure 2:**
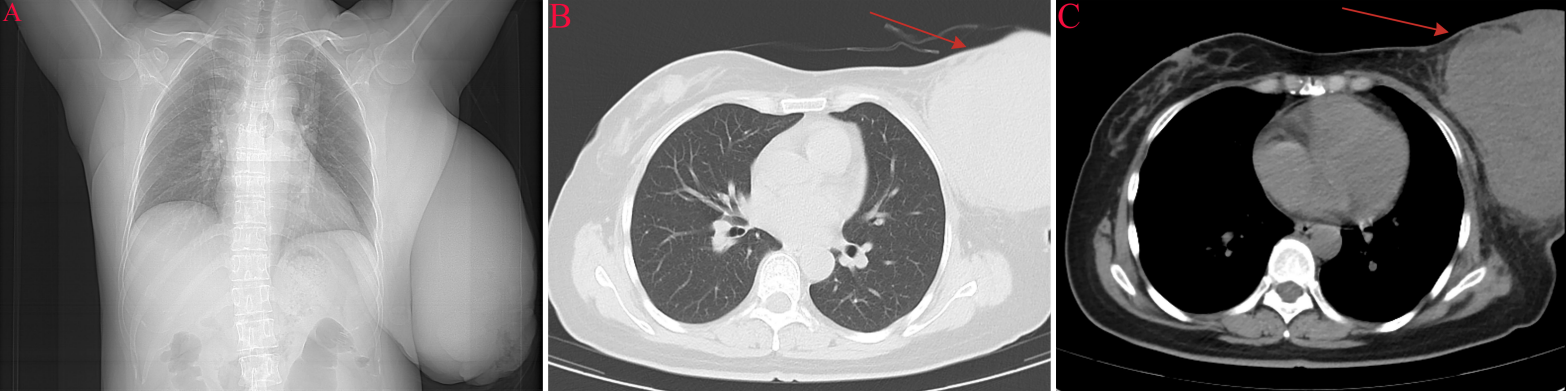
Preoperative chest imaging **(A–C)**. **(A)** Chest radiograph shows no obvious pulmonary metastasis or pleural effusion. **(B)** Axial CT (lung window) demonstrates a large left breast/anterior chest wall mass (arrow) without intrathoracic metastatic lesions. **(C)** Axial CT (mediastinal window) shows the mass with adjacent soft-tissue involvement (arrow).

**Figure 3:**
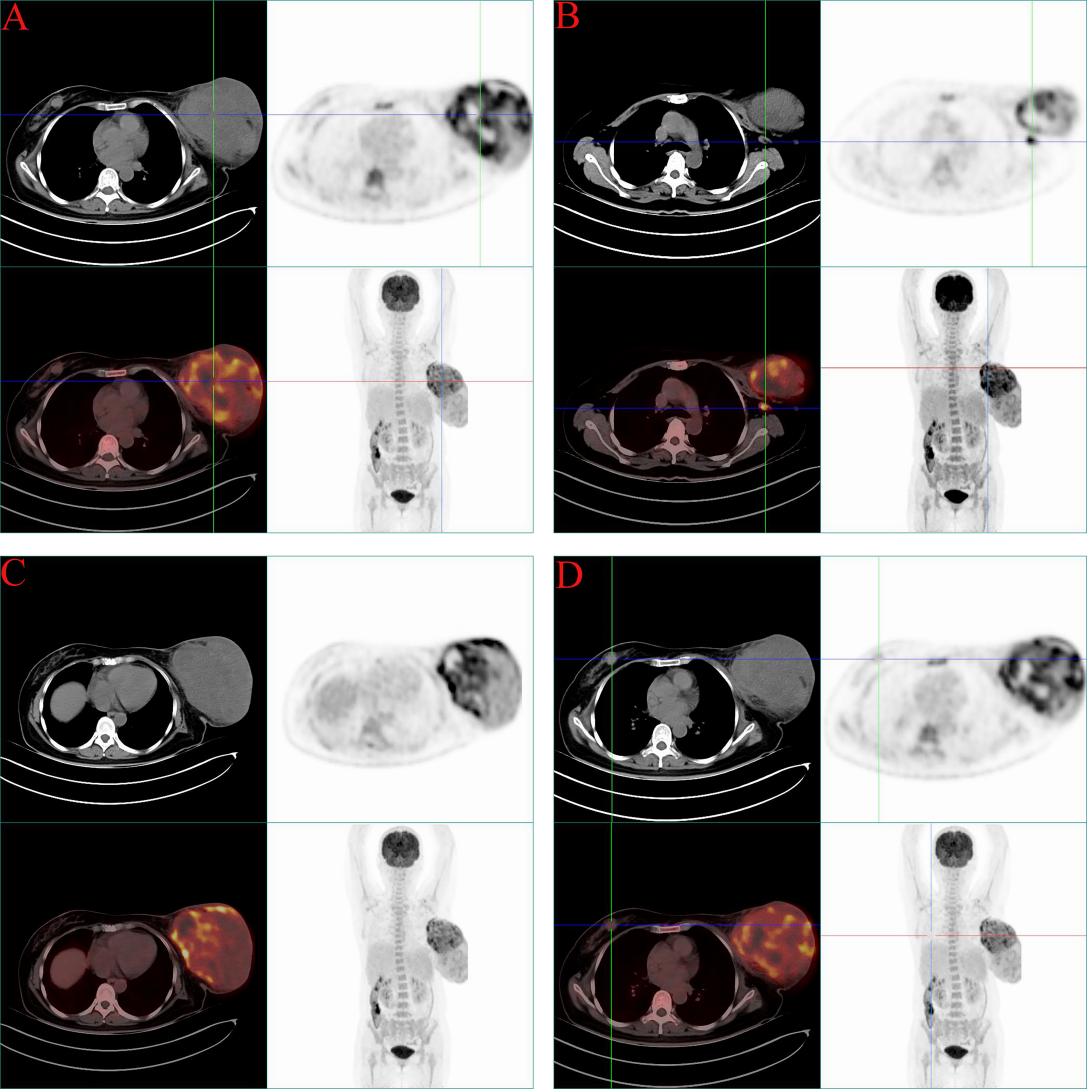
Representative ^18^F-FDG PET-CT images **(A–D)**. Axial CT, PET, and fused PET/CT images show heterogeneous intense FDG uptake within the left breast mass and markedly FDG-avid ipsilateral axillary lymph nodes. Whole-body maximum-intensity-projection images show no FDG-avid distant metastases.

Ultrasonography performed on May 19, 2025, demonstrated a hypoechoic nodule in the right breast at the 12 o’clock position (24.8 × 11.0 mm; BI-RADS 4A). The left breast contained a large heterogeneous mixed-echogenicity mass with increased vascularity and enlarged axillary lymph nodes (BI-RADS 0), warranting further evaluation. ^18^F-fluorodeoxyglucose positron emission tomography/computed tomography (^18^F-FDG PET-CT) was performed on May 20, 2025, for systemic assessment. A massive left breast soft-tissue lesion (16.9 × 12.3 × 23.4 cm) demonstrated heterogeneous intense FDG uptake (SUV_max_ 11.5). Multiple enlarged left axillary lymph nodes were identified (largest short-axis diameter 1.0 cm) with marked FDG avidity (SUV_max_ 9.0) and were interpreted radiologically as suspicious for nodal involvement; however, FDG uptake is not specific for malignancy and may also reflect reactive or inflammatory lymphadenopathy. A right breast nodule demonstrated mild uptake (SUV_max_ 1.7). No FDG-avid distant metastases were detected in the lungs, liver, or skeleton (**Figures 2A–C**, **3A–D**).

### Multidisciplinary decision and surgical management

A multidisciplinary team meeting was convened on May 21, 2025. The consensus was that the left lesion most likely represented a borderline phyllodes tumor with extensive necrosis and the right lesion a benign phyllodes tumor, with no definite distant metastasis. Although the FDG-avid axillary lymph nodes could represent false-positive inflammatory uptake, nodal metastasis could not be excluded with confidence. Because the giant ulcerated left breast mass and severe axillary edema were expected to impair tracer migration, sentinel lymph node biopsy was considered unreliable and was therefore not attempted. Surgery was performed on May 22, 2025. The operative time was 3.5 hours, estimated blood loss was 200 mL, and no transfusion was required. The right breast mass was excised, and intraoperative frozen section favored a benign fibroepithelial tumor consistent with benign phyllodes tumor; frozen-section margin assessment was negative, and permanent sections subsequently confirmed negative margins. A left total mastectomy was performed, including the nipple–areola complex and ulcerated skin, to achieve negative margins. A level I–II axillary dissection was undertaken because nodal metastasis could not be confidently excluded preoperatively. One drain was placed in the left axilla and one in the right breast; total postoperative drainage volumes were 150 mL and 50 mL, respectively, and both drains were removed 48 hours after surgery. Postoperatively, upper-extremity functional exercises were initiated to reduce the risk of lymphedema. Adjuvant treatment was planned to be determined according to the final pathology results.

The left breast total mastectomy specimen measured 26 cm × 18.5 cm × 11 cm, with a 20 cm × 16 cm tumor mass identified within the specimen. Microscopically, the tumor tissue was associated with extensive hemorrhage and coagulative necrosis. Histological examination revealed a biphasic fibroepithelial architecture with focal typical leaf-like projections protruding into glandular lumens. Stromal cells exhibited mild to moderate atypia with approximately 7 mitotic figures per 10 high-power fields (HPF). Based on the overall morphologic features, the tumor was diagnosed as a borderline phyllodes tumor, with the actual tumor dimensions measured at 19 cm × 14 cm × 11 cm. No tumor involvement was identified at the surgical margins, confirming negative margins; the closest margin was 8 mm at the deep margin. A total of 21 left axillary lymph nodes ranging from 0.5 to 1.5 cm were submitted for examination. Grossly, the nodes were regular in contour with intact capsules; microscopically, they showed follicular hyperplasia with prominent sinus histiocytosis and no evidence of tumor cell infiltration, consistent with inflammatory reactive hyperplasia.

The right breast lumpectomy specimen corresponded to the lesion at the 12 o’clock position as indicated by imaging examinations, with gross measurements of 2.5 cm × 1.1 cm × 0.8 cm (essentially consistent with the imaging result of 24.8 mm × 11.0 mm). Histologically, the tumor was diagnosed as a benign phyllodes tumor, characterized by bland stromal cells without atypia, rare mitotic figures, and absence of hemorrhage, necrosis, and stromal overgrowth. Intraoperative frozen-section margin assessment was negative, and postoperative paraffin sections confirmed negative margins; the closest margin was 5 mm at the peripheral margin.

**Figure 4:**
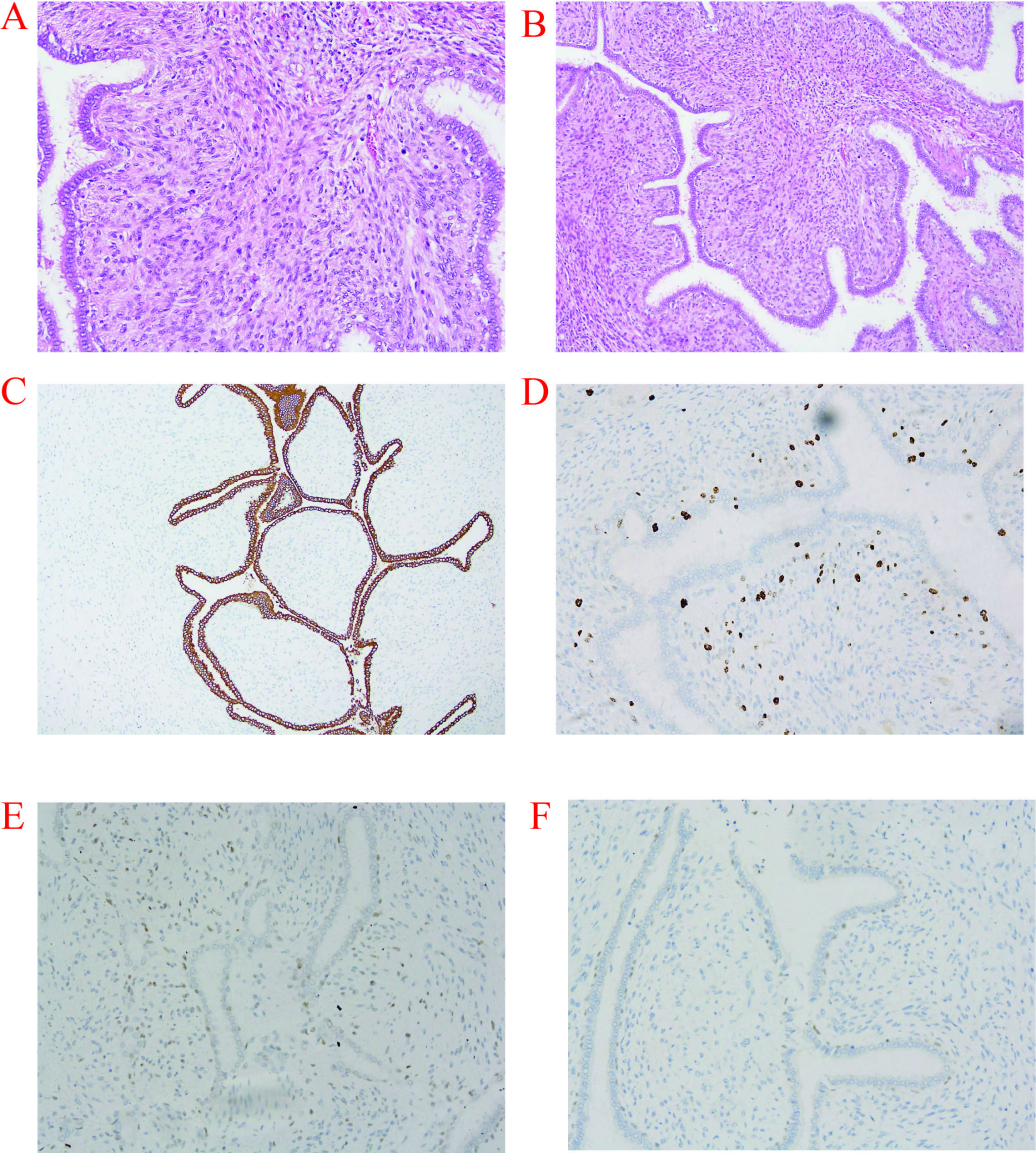
Histopathologic and immunohistochemical findings of the left breast phyllodes tumor. **(A)** Hematoxylin and eosin staining shows a biphasic fibroepithelial lesion with leaf-like architecture. **(B)** Higher-power view demonstrates increased stromal cellularity with mild-to-moderate atypia, consistent with borderline phyllodes tumor. **(C)** CK5/6 highlights the epithelial component. **(D)** Ki-67 shows a low proliferative index in stromal cells (~5%). **(E)** p53 shows weak/focal nuclear staining in stromal cells. **(F)** p63 highlights epithelial/myoepithelial nuclei and is negative in stromal cells. Original magnification: **(A)** ×100, **(B)** ×200, **(C–F)** ×100. Scale bars: **(A)** 200 μm, **(B)** 100 μm, **(C–F)** 200 μm.

Immunohistochemical (IHC) staining was performed on representative tissue sections of the left borderline phyllodes tumor, and hormone receptor testing was additionally undertaken. CK5/6 and p63 were evaluated in the epithelial/myoepithelial components, whereas Ki-67 and p53 were assessed in stromal hotspot areas. Both tumors were ER- and PR-, and the left tumor was also HER2-negative, arguing against hormone-dependent growth and indicating no established role for endocrine therapy. CK5/6 showed diffuse positivity in epithelial cells, and p63 showed nuclear positivity in epithelial/myoepithelial cell nuclei with no stromal staining. p53 showed focal weak nuclear expression in stromal hotspots, and the stromal Ki-67 proliferative index was approximately 5% (**Figure 4**). The discrepancy between the core-biopsy Ki-67 index (~2%) and the resection-specimen Ki-67 index (~5%) was interpreted as sampling-site related: the biopsy sampled peripheral stromal tissue, whereas the final specimen included hotspot areas from the tumor center. Taken together, the low stromal Ki-67 index and only focal weak p53 staining suggested relatively low stromal proliferative activity within the borderline category. A comprehensive postoperative assessment combining whole-body imaging and laboratory examinations revealed no evidence of distant metastasis.

### Postoperative course and follow-up

**Figure 5:**
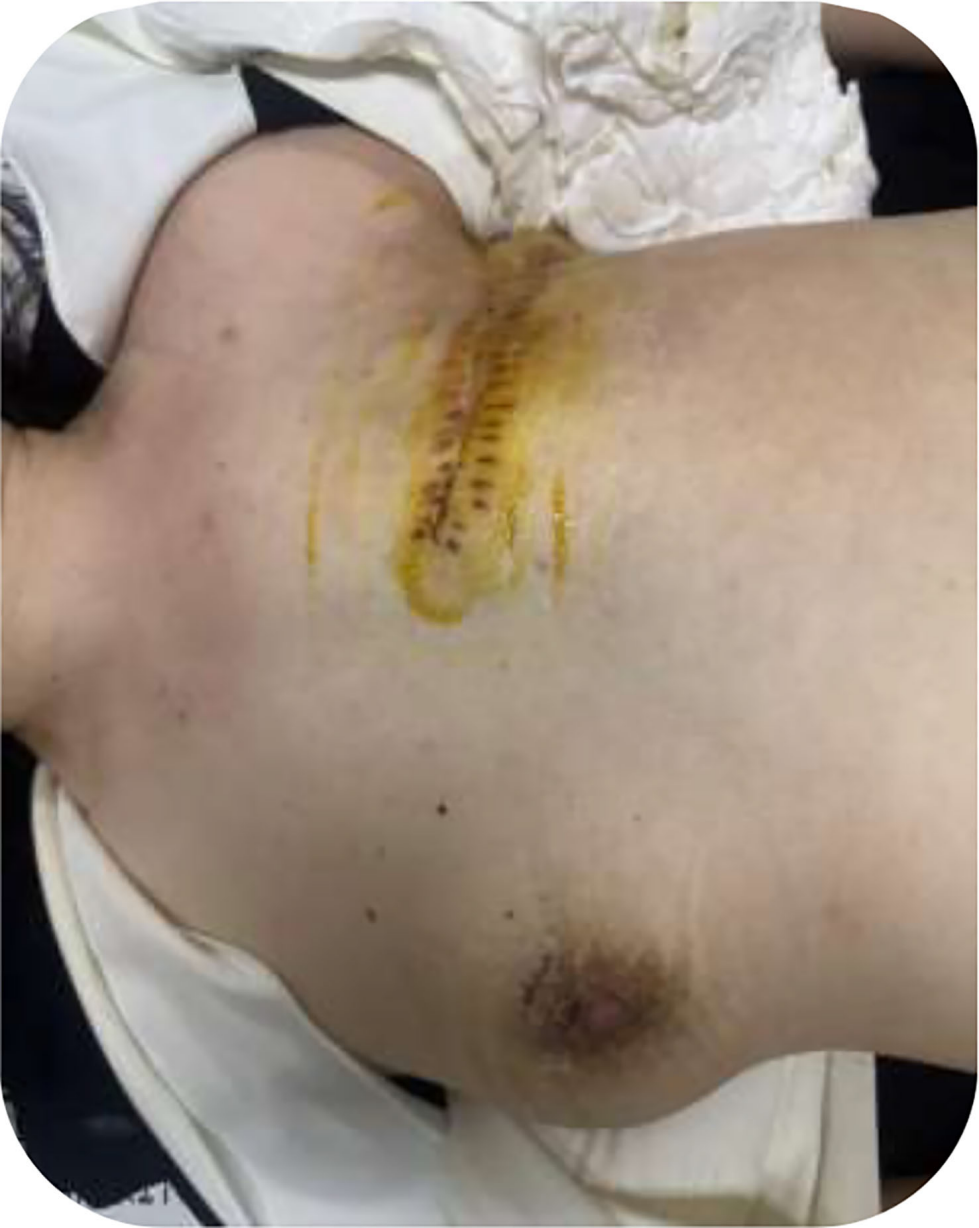
Postoperative clinical photograph. View after left mastectomy, demonstrating a well-approximated incision without early wound complications.

The patient recovered uneventfully and was discharged on June 3, 2025. Perioperative antimicrobial prophylaxis consisted of ceftriaxone sodium 2.0 g by intravenous drip once daily from 1 day before surgery through postoperative day 5, and no postoperative fever, wound infection, seroma, or other complications were observed. Postoperative anemia was managed with oral iron. At 1 month after surgery, CA125, CA15-3, and CA19–9 had all decreased to within the normal reference ranges. Given negative margins and the final benign/borderline histology, no adjuvant radiotherapy or chemotherapy was administered. Ultrasonographic surveillance every 6 months was planned, with particular attention to the left chest wall and axilla. In addition, the incidental PET/CT findings of a right thyroid nodule, right upper-lobe micronodule, and hepatic low-density lesion were scheduled for follow-up at 3 months with thyroid ultrasonography, chest CT, and abdominal ultrasonography, respectively. Key perioperative inflammatory and hematologic indices (WBC/NEU/PCT and Hb) are summarized in **Table 1**. The postoperative chest wall incision healed well without early wound complications (**Figure 5**).

## Discussion

A notable strength of the present report is that it documents synchronous bilateral phyllodes tumors with discordant histologic grades, namely a giant ulcerated borderline lesion in the left breast and a contralateral benign tumor detected only on imaging, thereby highlighting the importance of systematic bilateral evaluation in patients with clinically dominant unilateral disease. Beyond its rarity, this case also carries practical diagnostic implications, as it demonstrates that markedly FDG-avid axillary lymph nodes may represent false-positive findings and may consequently influence axillary surgical decision-making, underscoring the importance of careful clinicopathologic correlation to minimize potential overtreatment.

Phyllodes tumors (PTs) are uncommon fibroepithelial neoplasms of the breast with marked clinical and biologic heterogeneity, spanning indolent benign lesions to malignant tumors with metastatic potential. This heterogeneity complicates preoperative diagnosis, risk stratification, and treatment selection (1).According to the 2019 World Health Organization classification of breast tumors, PTs are fibroepithelial tumors arising from the intralobular/periductal stroma and are characterized histologically by stromal hypercellularity forming a distinctive leaf-like architecture. PTs are categorized as benign, borderline, or malignant based on a composite assessment of stromal cellularity and atypia, mitotic activity, stromal overgrowth, and the nature of the tumor border (2). PTs account for approximately 0.3%–1.0% of primary breast tumors and 2%–3% of fibroepithelial lesions (1).

The patient in this report was 44 years old, which falls within the commonly affected age range for PTs. Synchronous bilateral PTs remain exceptionally uncommon, with available evidence largely limited to case reports and small series (4). Proposed mechanisms, including host susceptibility, hormonal influences, and multicentric stromal proliferation, remain hypothetical (1). In the present case, the left-sided tumor reached 26 cm with skin ulceration, underscoring that PTs can attain massive size and produce advanced local disease (1, 8).

Accurate histologic grading is central to prognostication and treatment planning. Malignant PTs comprise a minority of cases and carry the greatest risk of distant metastasis, whereas benign and borderline PTs are predominantly associated with local recurrence and only infrequently with distant spread (1, 3, 8). Although histologic grading relies on multiple criteria, stromal overgrowth, hypercellularity/atypia, and mitotic activity remain among the most informative features for recognizing aggressive behavior (1, 8). In this case, the left tumor exhibited approximately 7 mitoses per 10 high-power fields together with increased stromal cellularity and mild-to-moderate stromal atypia, supporting a borderline classification without meeting commonly accepted thresholds for malignant PT. The absence of distant metastasis is consistent with this risk profile, although ongoing surveillance remains necessary.

Diagnosis requires integration of clinical presentation, imaging, pathology, and selected immunohistochemistry. Preoperative distinction between PTs and fibroadenomas, and between PT grades, remains challenging because of overlapping clinicoradiologic features and sampling limitations (1, 6). Core needle biopsy is generally preferred for preoperative evaluation, whereas fine-needle aspiration is less reliable for fibroepithelial lesions (1, 6). However, CNB may underestimate grade because of limited sampling, and definitive diagnosis often requires evaluation of the excision specimen (6). Immunohistochemical markers such as Ki-67 and p53 may support grading, but their values overlap across categories and should be interpreted as adjunctive rather than diagnostic criteria (10). In this case, the left lesion enlarged rapidly over 1 month and developed multiple ulcerations, whereas the contralateral lesion was clinically occult and detected only on imaging, illustrating the heterogeneous presentation and the risk of missing a synchronous lesion when symptoms are dominated by one side (4). Accordingly, comprehensive bilateral assessment should be considered when evaluating suspected PTs. Because the initial differential diagnosis included locally advanced breast carcinoma, primary breast sarcoma, and phyllodes tumor, CA-125, CA15-3, and CA19–9 were obtained as part of the preoperative diagnostic evaluation rather than as PT-specific biomarkers. Their transient elevation and subsequent normalization were interpreted as nonspecific findings associated with extensive tissue necrosis and systemic inflammation rather than with the biologic activity of PT itself.

Imaging modalities offer complementary information. Ultrasonography typically demonstrates a hypoechoic mass that may be heterogeneous, with increased vascularity and occasional cystic components (1). MRI, including radiomics-based approaches, has shown potential to improve discrimination between PTs and fibroadenomas and may assist in grading in selected scenarios, particularly for large or clinically concerning tumors (9). ^18^F-FDG PET-CT may be used for systemic assessment in suspected malignant disease; however, FDG uptake is not specific for malignancy, and inflammatory change or necrosis can produce false-positive findings, including FDG-avid axillary lymph nodes (1, 7). In the present case, PET/CT had practical clinical value because it provided whole-body assessment of a massive clinically aggressive-appearing breast lesion and identified markedly FDG-avid ipsilateral axillary nodes that required further consideration during preoperative evaluation. At the same time, this case also illustrates an important limitation: axillary nodal hypermetabolism should not be equated with metastatic involvement in the absence of pathologic confirmation.

To move beyond a purely descriptive interpretation, the FDG uptake pattern in this case can be understood as the combined result of stromal proliferative activity, inflammatory activation, and intratumoral heterogeneity. Phyllodes tumor grading is fundamentally based on stromal cellularity, atypia, mitotic activity, stromal overgrowth, and tumor borders, and the left-sided lesion in our patient showed borderline histology with increased stromal cellularity and approximately 7 mitoses per 10 high-power fields (1). In general, FDG accumulation reflects glucose transporter-mediated cellular uptake and subsequent hexokinase-mediated intracellular trapping, and prior work in breast carcinoma has shown a correlation between intratumoral FDG uptake and GLUT1 expression (21, 22). Accordingly, the intense uptake of the primary left breast tumor may partly reflect the metabolic demand of its hypercellular and proliferative stromal component rather than tumor size alone.

At the level of the reactive lymph nodes, the increased ^18^F-FDG uptake was likely driven by a different mechanism. Extensive ulceration and necrosis on the surface of a giant tumor may induce both severe local secondary infection and a systemic sterile inflammatory response. In our patient, this interpretation was supported by the markedly elevated C-reactive protein 165.7mg/L and interleukin-6 61.9pg/mL levels at admission. Under the influence of these inflammatory mediators, activated neutrophils and macrophages may accumulate in the draining axillary lymph nodes and increase their glucose utilization to support chemotaxis and phagocytosis. As a result, reactive nodal tissue may show substantial FDG accumulation, thereby mimicking metastatic spread on PET/CT. This mechanism is consistent with previous reports highlighting reactive lymphadenitis as a source of false-positive nodal uptake (20, 23).The heterogeneous uptake pattern in the present case was likely shaped by a mixture of viable stromal tissue, hemorrhage/necrosis, and inflammatory change, and the marked axillary nodal avidity should therefore be interpreted in light of both tumor biology and the accompanying inflammatory microenvironment rather than being assumed to represent metastatic spread.

Crucially, the synchronous bilateral presentation of these tumors provided a rare *in vivo* opportunity to examine whether metabolic imaging might reflect histologic heterogeneity. In the present case, the left-sided lesion was a borderline PT with increased stromal cellularity, mild-to-moderate stromal atypia, and approximately 7 mitoses per 10 high-power fields, whereas the right-sided lesion was a benign PT characterized by bland stromal cells, rare mitotic activity, and absence of hemorrhage, necrosis, and stromal overgrowth (2, 10). These histologic differences were paralleled by a striking metabolic discordance on PET/CT: the left-sided borderline tumor demonstrated intense uptake (SUV_max_ 11.5), whereas the right-sided benign lesion showed only mild uptake (SUV_max_ 1.7). Although PET/CT cannot reliably replace histopathologic grading, this contrast suggests that FDG uptake may partly reflect stromal proliferative activity and heterogeneity in discordant fibroepithelial lesions. Thus, despite its limited specificity for inflammatory nodal findings, PET/CT may still provide partial insight into the metabolic and pathologic heterogeneity of the primary tumors themselves when interpreted together with histopathology (4, 7).

Given these observations, it is also useful to place the present case within the context of recent advances in molecular imaging. Fibroblast activation protein inhibitor (FAPI) PET/CT has emerged as a promising stromal-targeted imaging approach because fibroblast activation protein is highly expressed in cancer-associated fibroblasts and in the stromal compartment of many epithelial malignancies, including breast cancer (24). Recent reviews suggest that FAPI-based tracers may improve lesion conspicuity, staging, and assessment of disease extent in breast malignancies by targeting activated stromal fibroblasts rather than glucose metabolism alone (24). From a conceptual standpoint, this may also be relevant to phyllodes tumors, which are fibroepithelial neoplasms characterized by a prominent stromal component (10, 24). Nevertheless, current evidence also indicates that FAPI uptake is not entirely tumor-specific and may be seen in inflammatory and fibroproliferative conditions as well (24, 25). Therefore, we do not suggest that FAPI PET/CT would have definitively resolved the nodal ambiguity in the present case; rather, we propose that it may represent a useful complementary direction for future investigation in stroma-rich tumors such as PT, particularly in scenarios where conventional FDG imaging is difficult to interpret because of ulceration, necrosis, and intense inflammatory activation (24, 25).

Against this background, Surgery remains the cornerstone of phyllodes tumor management. The roles of adjuvant radiotherapy and systemic therapy remain controversial and are typically individualized based on grade, tumor size, margin status, and recurrence risk (1, 11). The principal surgical goal is complete excision with negative margins. Although the optimal margin width remains debated, multiple studies emphasize that margin status is more important than a specific numeric width, with more stringent attention to margins generally warranted in borderline and malignant disease (5, 12, 16). Mastectomy may be required for giant tumors, tumors replacing most of the breast, or situations in which negative margins cannot be reasonably achieved with breast-conserving surgery (1, 5). In giant PTs (>20 cm), breast-conserving surgery often fails to reliably achieve adequate margins because of extensive parenchymal replacement and skin involvement; in such scenarios, upfront total mastectomy is frequently the most dependable strategy to achieve negative margins and may potentially spare selected patients from adjuvant radiotherapy after inadequate-conservation attempts (19).In the present case, mastectomy was necessary for the left-sided 26-cm ulcerated tumor, whereas the smaller right-sided lesion was managed with excision; both achieved negative margins.

Axillary management remains an area of uncertainty. Phyllodes tumors predominantly metastasize hematogenously; nodal metastasis is rare, and routine axillary dissection is generally not recommended in the absence of pathologic confirmation (1, 16). However, when imaging suggests nodal involvement, especially with marked FDG avidity, tissue confirmation should be pursued when feasible to avoid overtreatment. In this case, ALND was performed because metastatic disease could not be excluded preoperatively and sentinel lymph node biopsy was not technically feasible due to severe axillary edema; final pathology demonstrated reactive hyperplasia (0/21), underscoring the importance of integrating imaging with histology to minimize unnecessary axillary surgery. Evidence supporting sentinel lymph node biopsy in PT remains limited (1, 16).

Adjuvant radiotherapy may reduce local recurrence in selected borderline or malignant PTs, particularly after breast-conserving surgery, in large tumors, or when margins are close or positive; however, a consistent survival benefit has not been demonstrated (11, 14, 15). Given the negative margins and the overall clinicopathologic profile in this patient, adjuvant radiotherapy was omitted. Chemotherapy is not routinely recommended and is generally reserved for unresectable, recurrent, or metastatic malignant disease; endocrine therapy has no established role (1, 11, 14).

Prognosis depends on histologic grade, margin status, tumor size, and proliferative activity. Reported local recurrence rates vary across cohorts; commonly cited ranges are approximately 10%–17% for benign PT, 14%–25% for borderline PT, and 23%–30% for malignant PT (3, 13, 17, 18). Malignant PTs carry the highest risk of distant metastasis, most often involving the lungs and bone (1, 8, 18). Most local recurrences occur within the first 2–3 years, and positive margins are among the strongest predictors (3, 12, 13). Accordingly, follow-up should be risk-adapted and focus on clinical assessment and imaging surveillance. In this case, ultrasonographic surveillance every 6 months was planned, which is appropriate for early detection of local recurrence during the period of highest risk.

Patient perspective. The patient reported relief upon learning that the axillary lymph nodes were benign despite the initial concern for metastasis. She expressed satisfaction with the cosmetic outcome of the right breast and reported adapting well following the left mastectomy.

Conclusion and clinical reflection. Synchronous bilateral phyllodes tumors are distinctly uncommon and pose diagnostic and management challenges because of overlapping clinicoradiologic features with other fibroepithelial lesions and limited high-level evidence guiding care. In this case, a giant ulcerated borderline tumor in the left breast and a benign contralateral tumor in the right breast were accompanied by false-positive axillary ^18^F-FDG uptake, which was ultimately explained by inflammatory reactive hyperplasia rather than metastatic disease. Comprehensive bilateral assessment, careful interpretation of FDG-avid axillary nodes, and individualized surgical planning were essential to appropriate management. This case also underscores the value of correlating imaging, pathology, and operative findings to avoid overtreatment.

## Data Availability

The original contributions presented in the study are included in the article/supplementary material. Further inquiries can be directed to the corresponding author/s.
